# Community–Academic Partnerships: A Report on the COVID Support Our Schools (SOS) Initiative

**DOI:** 10.3390/children9121868

**Published:** 2022-11-30

**Authors:** Carmela Rocchetti, Srividya Naganathan, Michal Divney, Joshua Josephs, Harpreet Pall

**Affiliations:** 1 Department of Internal Medicine, Hackensack Meridian School of Medicine, Nutley, NJ 07110, USA; 2 Department of Pediatrics, Hackensack Meridian School of Medicine, K Hovnanian Children’s Hospital, Jersey Shore University Medical Center, Neptune, NJ 07753, USA; 3 Human Dimension and Community Programs, Hackensack Meridian School of Medicine, Nutley, NJ 07110, USA

**Keywords:** academic health departments, community–academic partnership, educational disparities, medical student engagement

## Abstract

The SARS-CoV-2 pandemic disrupted the delivery of education in our school systems and exacerbated pre-existing health and educational disparities. School administrators and staff from underserved communities struggled with continuously changing medical guidance, ensuring equitable education by virtual platforms, and re-opening schools safely. The Hackensack Meridian School of Medicine (HMSOM) partnered with statewide resource-limited school districts to create the COVID Support Our Schools (SOS) initiative. This consisted of a physician advisory board, medical student task forces, and community leaders. Numerous sessions were hosted by the COVID SOS team to answer questions, address community needs, and carry out initiatives to meet identified needs. Surveys, including Likert scale questions and open-ended feedback, were administered to all participating school districts. In total, 100% of respondents strongly agreed or agreed that the COVID SOS program was dedicated to community needs and provided timely support with necessary resources. Open-ended feedback described that the K-12 school districts valued our partnership as well as found the opportunity to engage with other districts informative and rewarding. The partnership between our academic medical institution and community school districts is mutually beneficial, providing an opportunity for medical student service learning as well as imparting medical expertise in times of need.

## 1. Introduction

The severe acute respiratory syndrome coronavirus 2 (SARS-CoV-2) pandemic has not only been challenging for the medical community but has caused chaos and disruptions in our kindergarten through 12th grade (K-12) school systems [1]. Many schools remained closed for in-person learning for the 2020–21 academic year, and school officials innovated to implement virtual learning platforms. Unfortunately, the lack of in-person interactions between teachers and students led to disengagement and further highlighted health and educational inequities. School districts struggled to keep up with changing guidelines, staff education, and vaccine education and policies. These challenges due to the pandemic led to stunted behavioral and socioemotional development in children [2,3]. This dramatic toll on schools also affected students’ nutrition and attendance. Students on special education plans were uniquely impacted due to the loss of instructional time [4]. Not only were the students impacted, but teachers sought mental health support and had higher rates of absenteeism.

Academic health departments or community institutional partnerships refers to the collaboration between academic medical institutions and public/community departments [5,6]. Community engagement has shown to be of mutual benefit; community departments gain the expertise and advice from the medical institution while the academic institution gains the benefit of learner engagement with the community [7]. During the SARS-CoV-2 pandemic, medical institutions successfully partnered with school districts to provide much-needed support and guidance [8,9]. Although some clinical activities of medical students were suspended with rising cases of coronavirus disease 2019 (COVID-19), the pandemic provided unique and innovative ways for medical students to engage in community support including with local school systems [10,11].

At the Hackensack Meridian School of Medicine (HMSOM), we collaborated with our local K-12 school districts and implemented the COVID Support Our Schools (SOS) initiative. Pre-existing well-established community partnerships were essential for rapidly identifying and responding to the needs of the community during this health crisis. This highlights the value for medical schools to develop strong community partnerships, which proved to be instrumental in a healthcare crisis.

The objectives of this report are to illustrate our mutually beneficial community–academic partnership and review quantitative and qualitative feedback about the program from our community partners.

## 2. Materials and Methods

**Figure 1:**
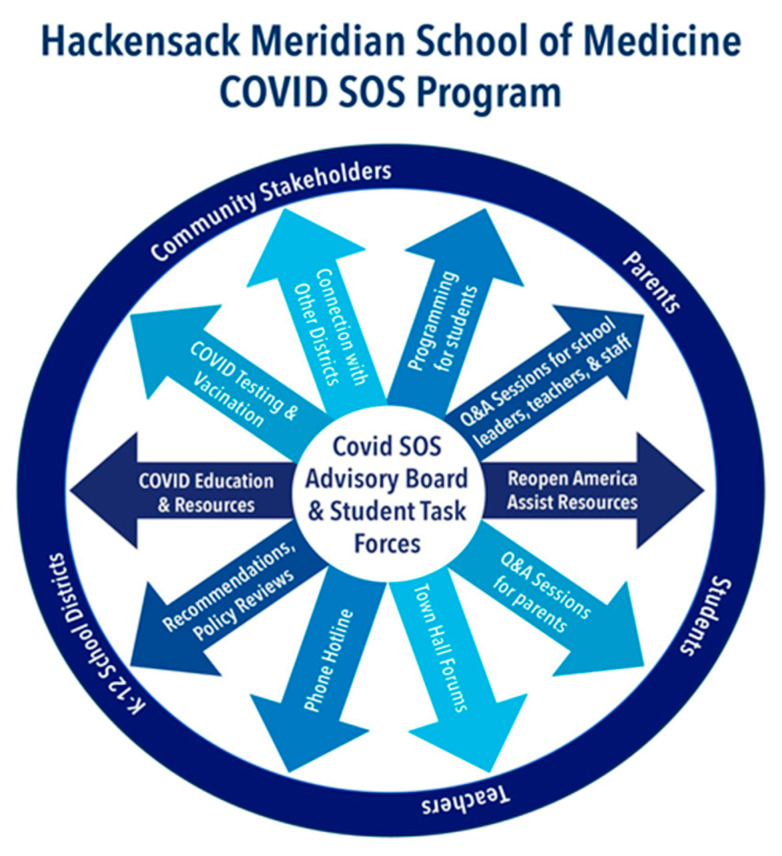
The COVID SOS Program.

The COVID SOS initiative was developed as part of the Human Dimension Course at HMSOM. This curriculum was built upon prior established philosophies of community-engaged medical education (CEME) which “underscores a relationship between the community served and medical school that is both interdependent and reciprocal” [12,13]. Fundamental to the concept of CEME is that students can learn about the community experientially, while the community is also engaged in student education, thus mutually providing solutions to the community’s health challenges. CEME has been described in medical education and other health professions as a method to effectively engage the community in health education and to engage the learners in addressing the needs of the communities. Through the required Human Dimension course, student teams are longitudinally matched to community partners to identify needs and develop targeted health interventions. As part of the academic restructuring that took place during the pandemic, HMSOM developed the COVID SOS initiative as a means of aligning student learning objectives and community needs. Because of already established strong community relationships, this initiative was able to form quickly and effectively. The SOS program consisted of two main components: the COVID SOS Advisory Board and 20 student task force groups (Figure 1).

K-12 school districts were invited to join the program based on their preexisting relationship with HMSOM and the Human Dimension Course. In total, 11 school districts and one Boys and Girls Club representing 138 individual schools across a broad geographic region in New Jersey, from historically underserved areas with students from marginalized populations, were enrolled in the program. Community needs were identified during town hall, recurring meetings, individual conversations, and correspondence.

The COVID SOS Advisory Board consisted of an interprofessional committee of clinical physician faculty and a community engagement team. Twelve faculty members representing diverse specialties including pediatrics, internal medicine, family medicine, infectious disease, library sciences, and emergency medicine worked alongside a team of two social workers, one community engagement specialist, and four community liaisons.

Since the spring of 2020, the COVID SOS Advisory Board has hosted regular Question and Answer (Q&A) sessions for school leaders at various intervals ranging from weekly to every 3 months based on local COVID-19 trends. A total of 24 sessions, each 60–90 minutes long, were conducted via video conferencing. Each session had a topic of focus which was pre-determined by community member vote during the previous session. During the virtual sessions, community partners and faculty were placed into breakout groups to allow for individualized support; groups would then report back to share challenges and solutions in a discussion with the full group. Session topics included: COVID Guidelines, Staffing and Operational Challenges, Child Abuse and Neglect during COVID-19, Masking, Planning for Vaccinations, Education Disparities, Unique Challenges of Remote Learning, and the Mental Health impact of COVID-19 on Communities. In between Q&A sessions, school districts had direct access to faculty to answer questions specific to their school in a timely manner.

The COVID SOS Student Task Force component of the initiative matched groups of eight preclinical medical students supervised by one clinical faculty with school districts and specific schools to support leadership on any COVID-19-related challenges the school(s) elected. The task forces met with their school partners approximately every 4–6 weeks via video conferencing for approximately 60 min, during which the schools outlined challenges and tasks for which the students could help research and create solutions. Examples of task force areas of focus included: Translating and Operationalizing COVID-19 Guidelines, Virtual Interactive COVID-19 Educational Series for Elementary Students, COVID-19 Vaccine Q&A for Students, Parents and School Staff, Developing and Hosting Mental Health and Wellness Programs for Middle School Students, and Programming to Improve Digital Literacy. Both the COVID SOS Advisory Board and the Student Task Forces were supported by the HMHSOM Community Programs Team.

The study survey was approved by the Institutional Review Board (IRB) committee at Hackensack Meridian Health and designed to evaluate the success of the program as well as identify ongoing needs. Questions were structured to provide both quantitative and qualitative feedback. The survey was administered to all school personnel who participated in the program. The school district and job category were collected and this information was then de-identified, with limited staff having access to the identifiable data.

Qualitative response themes from the survey included:

Informative: Obtaining real-time interpretation of COVID-19 guidelines from experts as well as information sharing among school districts and their challenges and responses.

Relationship Building: Ability to develop long-lasting relationships with both the medical community and other school districts.

Medical Student Involvement: Contributions from medical students to individualized needs of the schools.

## 3. Results

**Figure 2:**
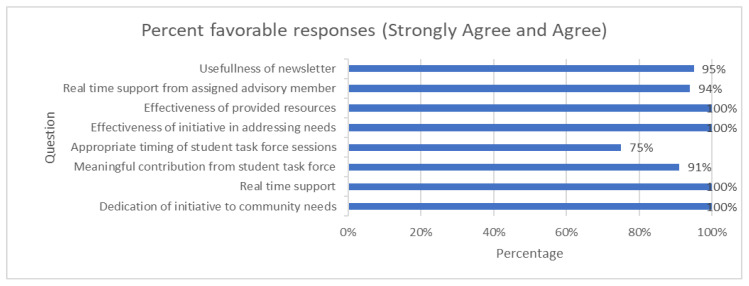
Survey responses with percent strongly agree and agree.

All of the schools who were invited to participate in the program accepted. There was an 83% response rate to the survey sent to the participating school districts to evaluate our COVID SOS initiative. The survey questions explored the impact, effectiveness, and timely support of the COVID SOS initiative as well as addressing community needs and providing resources. The responses were scored on a 5-point Likert scale from ‘strongly agree’ to ‘strongly disagree’ as well as open-ended questions for comments regarding the initiative. ‘Strongly agree’ and ‘agree’ responses were grouped as favorable as shown in Figure 2. Qualitative responses from open-ended feedback are shown in Table 1.

Open-ended feedback regarding the most helpful aspects of the initiative revealed that the discussions were very informative and that having medical expertise outside the districts’ resources was incredibly valuable. The opportunity to connect with other school districts and to build relationships was also a prominent theme. Lastly, comments pointed to the valuable contributions that the medical students made for the districts’ work around COVID-19. In total, 100% of respondents reported that they would recommend partnering with a local medical school on a program like this to other school districts.

## 4. Discussion

This program highlights the critical importance of established community partnerships between academic medical institutions and community stakeholders to provide invaluable assistance during a time of need, such as a global pandemic.

Community stakeholders can often provide deep insight and background, thereby increasing research relevance and feasibility [14]. It is critically important for academic medical institutions to engage these community partners for direct input into initiatives and research studies. This improved collaboration can ensure the translation of research results into a real-world setting.

In addition, involving community stakeholders such as school districts can decrease the marginalization of communities that have historically not been involved in research settings. The approach of treating the community stakeholder as an equal partner engenders trust and an optimal collaboration.

These partnerships are mutually beneficial by providing medical students important real-life experiences to develop advocacy and social determinants knowledge and skills, while also providing the community with guidance based on medical expertise. Medical students are in a unique position to mobilize effectively and quickly during a healthcare crisis.

The unique characteristics of this partnership facilitated the collaborative process and promoted engagement amongst all parties. The medical students, physicians, and school leaders rated this initiative highly. Together, the teams were able to display an agile response to a rapidly changing environment.

Limitations: Our sample may not be representative of the state of New Jersey or the entire United States. Although the school staff found the information presented in our sessions useful, we were not able to measure outcomes such as vaccination rates of school personnel and students, effectiveness of school policies in infection prevention and control, and student performance during the pandemic.

## 5. Conclusions

Our initiative can serve as a framework for other institutions to establish a community–academic partnership. Such initiatives and community involvement can continue beyond the pandemic to address ongoing healthcare concerns and challenges such as vaccine hesitancy, health curriculum, and sports safety.

## Figures and Tables

**Table 1:** SOS Survey Qualitative Responses.

What Was the Most Helpful or Impactful Aspect of the Overall SOS Initiative?
Working with the student task forces to develop content for our staff Meeting with other school districts to discuss the pandemic and its effects on our students, teachers, families and community as a whole Sharing information with surrounding districts. Interacting with medical experts, looking for solutions to our issues Collaboration with other districts and medical professionals Informative and relationship building It was wonderful having access to real-time information from experts in our area Also, having the medical students directly interact with our students Having a medical partner outside of the “district” resources Working as a partnership to help us with COVID-19 (parent forums, questions, medical students input and support in creation of book) Outstanding resources and facts that we can apply to our schools and community Being able to participate in the sessions and have our questions answered Discussions were very informative They shared with us useful infographics and are willing to come to our school and meet with students that may benefit from their presence and attention. We are trying to figure out what is the best time to do that Open discussion with Garfield School District, having advice and discussing ongoing activities and issues Overall, the sessions were very well organized and quite informative. The medical school’s physicians and support staff provided excellent guidance and resources. Thank you so much for everyone’s comprehensive delivery of facts and overall medical research. Truly enjoyed the discussions and resources. The participation and support were very much appreciated.
